# Natural Transformation of *Riemerella columbina* and Its Determinants

**DOI:** 10.3389/fmicb.2021.634895

**Published:** 2021-03-03

**Authors:** Li Huang, Mafeng Liu, Dekang Zhu, Li Xie, Mi Huang, Chen Xiang, Francis Biville, Renyong Jia, Shun Chen, Xinxin Zhao, Qiao Yang, Ying Wu, Shaqiu Zhang, Juan Huang, Xumin Ou, Sai Mao, Qun Gao, Di Sun, Bin Tian, Mingshu Wang, Anchun Cheng

**Affiliations:** ^1^Institute of Preventive Veterinary Medicine, Sichuan Agricultural University, Chengdu, China; ^2^Research Centre of Avian Disease, College of Veterinary Medicine, Sichuan Agricultural University, Chengdu, China; ^3^Key Laboratory of Animal Disease and Human Health of Sichuan Province, Chengdu, China

**Keywords:** *Flavobacteriaceae*, *R. columbina*, *Flavobacterium johnsoniae*, natural competence, horizontal gene transfer

## Abstract

In a previous study, it was shown that *Riemerella anatipestifer*, a member of *Flavobacteriaceae*, is naturally competent. However, whether natural competence is universal in *Flavobacteriaceae* remains unknown. In this study, it was shown for the first time that *Riemerella columbina* was naturally competent in the laboratory condition; however, *Flavobacterium johnsoniae* was not naturally competent under the same conditions. The competence of *R. columbina* was maintained throughout the growth phases, and the transformation frequency was highest during the logarithmic phase. A competition assay revealed that *R. columbina* preferentially took up its own genomic DNA over heterologous DNA. The natural transformation frequency of *R. columbina* was significantly increased in GCB medium without peptone or phosphate. Furthermore, natural transformation of *R. columbina* was inhibited by 0.5 mM EDTA, but could be restored by the addition of CaCl_2_, MgCl_2_, ZnCl_2_, and MnCl_2_, suggesting that these divalent cations promote the natural transformation of *R. columbina*. Overall, this study revealed that natural competence is not universal in *Flavobacteriaceae* members and triggering of competence differs from species to species.

## Introduction

Naturally competent bacteria can actively take up naked DNA from their environment and integrate it into the genome, which is called natural transformation (Mell and Redfield, 2014). As one of the three horizontal gene transfer mechanisms, natural transformation facilitates bacterial acquisition of virulence genes and antibiotic-resistant cassettes to help bacteria adapt to the environment (Wiedenbeck and Cohan, 2011; Seitz and Blokesch, 2013a). Natural transformation was first discovered in *Streptococcus pneumoniae* in 1928 (Griffith, 1928). Currently, at least 83 species have been found to have natural competence (Johnston et al., 2014; Liu et al., 2017).

In Gram-positive and Gram-negative bacteria, there are different mechanism to take up DNA. Naturally competent Gram-negative bacteria, such as *Neisseria* species and *Haemophilus influenzae*, use type IV pili (T4P) to take up exogenous double-stranded DNA (dsDNA), in contrast to *Helicobacter pylori*, which uses a type IV secretion system (T4SS) (Hofreuter et al., 2000), and *Campylobacter jejuni*, which uses a type II secretion system (T2SS) (Wiesner et al., 2003) to take up exogenous dsDNA. Gram-positive bacteria, such as *S. pneumoniae* and *Bacillus subtilis*, use a competence pseudopilus, a structure similar to T4P, to take up dsDNA (Hahn et al., 2005). Once dsDNA is transported across the outer membrane in Gram-negative bacteria or the peptidoglycan layer in Gram-positive bacteria, dsDNA is degraded to single-stranded DNA (ssDNA) and transported through the pore protein ComEC into the cytoplasm (Johnston et al., 2014). Internalized ssDNA is presumably bound by DNA-processing protein A (DprA), which recruits the recombinase RecA to mediate homologous recombination by facilitating strand exchange (Johnston et al., 2014; Huang et al., 2019). At present, the natural transformation of *H. influenzae* in *Pasteurellaceae*, *Vibrio cholerae* in *Vibrionaceae*, and *S. pneumoniae* in *Streptococcaceae* are well studied (Redfield et al., 2006; Blokesch, 2012; Johnston et al., 2014). Among the six genera of *Pasteurellaceae*, only the genera *Actinobacillus* and *Haemophilus* are naturally competent (Redfield et al., 2006). In *Streptococcaceae*, both *Streptococcus* and *Lactococcus* show natural competence (Griffith, 1928; Dalia et al., 2017). Within the genus *Haemophilus*, when *H. influenzae* is transferred from rich medium to defined competence medium (M-IV) or the cell culture reaches stationary phase, it becomes naturally competent (Herriott et al., 1970; Redfield, 1991). However, natural transformation of *Haemophilus parasuis* was readily induced by nutrient-rich medium (Zhang et al., 2015; Li et al., 2016). All information suggests that the occurrence of natural transformation is different among bacteria, even within the same genus.

In a previous study, it was shown that one member of the *Flavobacteriaceae* family, *Riemerella anatipestifer* (*R. anatipestifer*, RA), which causes septicemic diseases in ducks, geese, turkeys, and other birds (Huang et al., 2017), is naturally competent (Liu et al., 2017). However, whether other *Flavobacteriaceae* species are also naturally competent and under which condition natural transformation is induced remains unknown. Here, *Flavobacterium johnsoniae* (*F. johnsoniae*), a common soil and aquatic bacterium (McBride, 2004), and *Riemerella columbina* (*R. columbina*), widely distributed species among pigeon populations (Rubbenstroth et al., 2013), were selected as models to explore the occurrence of natural transformation and its influencing factors.

## Materials and Methods

### Bacterial Strains, Primers, and Growth Conditions

*Riemerella columbina* and *Flavobacterium johnsoniae* were purchased from the Culture Collection of the University of Gothenburg (CCUG) and the China General Microbiological Culture Collection Center (CGMCC), respectively. The bacterial strains and primers used in this study are listed in Table 1. The culture conditions for *R. columbina* and *F. johnsoniae* were identical to those used for *R. anatipestifer* described in a previous study (Huang et al., 2019). Briefly, *R. columbina* was cultured in GC broth (GCB) medium with shaking or GCB agar plates and LB plates supplemented with 5% sheep blood (blood plates) at 37°C, however, *F. johnsoniae* was cultured in GCB medium with shaking or GCB plates at 25°C. When required, erythromycin was added into the medium at a final concentration of 1 μg/ml for *R. columbina* and 50 μg/ml for *F. johnsoniae*.

**TABLE 1:** Strains and primers used in this study.

**Strain**	**Genotype or description**	**Source**
*F. johnsoniae*	*F. johnsoniae* ATCC 17061	CGMCC
*R. columbina*	*R. columbina* CCUG 47689	CCUG
*R. columbina*Δ*C237_RS0105470*::*Erm*	*R. columbina*Δ*C237_RS0105470*, Erm^*R*^	This study
*R. anatipestifer* ATCC11845	*R. anatipestifer* ATCC11845, Kan^*R*^	This study
*R. anatipestifer* CH-1	*R. anatipestifer* ATCC11845, Kan^*R*^, Erm^*R*^	This study

**Primer**	**Sequence**	**Source**

Up(gldH) P1	TAGCCGGACAATGTGGTAAACTAAAATGCT	*F. johnsoniae*
Up(gldH) P2	GACTGGAAAGTGGTTTTTTGTGATAATTATAGGTTTT	*F. johnsoniae*
Erm(gldH) P1	ATAATTATCACAAAAAACCACTTTCCAGTCTTACGAA	*R. anatipestifer* CH-1
Erm(gldH) P2	ATAACTATTTTTCGACTTTGAACTACGAAGGATGAAA	*R. anatipestifer* CH-1
Down(gldH) P1	GTAGTTCAAAGTCGAAAAATAGTTATGGCTGCTAAAA	*F. johnsoniae*
Down(gldH) P2	TTTTGAGAAATAGGTTTGTGCTGCTGAGCT	*F. johnsoniae*
RC-Up P1	CCCACATAGTTTGCGTAGAGATTATTTTGCC	*R. columbina*
RC-Up P2	CTGGAAAGTGGTAGAAACAAATGTAATAAATTTTTCG	*R. columbina*
RC-Erm P1	TTATTACATTTGTTTCTACCACTTTCCAGTCTTACGA	*R. anatipestifer* CH-1
RC-Erm P2	GATTTTATAGCGTCGACTTTGAACTACGAAGGAT	*R. anatipestifer* CH-1
RC-Down P1	TAGTTCAAAGTCGACGCTATAAAATCACGATTAAAA	*R. columbina*
RC-Down P2	TGTCGGATTTCCCTTGTGGGTCAAA	*R. columbina*
RC-16S rRNA P1	ATGGAATTAATACAGCAACATTTTG	*R. columbina*
RC-16S rRNA P2	TCAAATATGCCCTTTAGAAAGGTA	*R. columbina*
*C237_RS0105470* P1	ATGAATACTGAAGAAATTTTATATGCTA	*R. columbina*
*C237_RS0105470* P2	AATTGAATATAAGCGTCCCGA	*R. columbina*

### Preparation of Donor DNA

The homologous gene of *dprA* (*C237_RS0105470*) in *R. columbina*, which protects ssDNA and loads RecA to facilitate homologous recombination (Mirouze et al., 2013), and the gliding motility gene *gldH* in *F. johnsoniae* were selected as targeted deletion gene, since they are not essential for the growth of bacteria and can be deleted (McBride et al., 2003; Hovland et al., 2017; Huang et al., 2019). Donor DNA was composed of upstream of target gene, an antibiotic resistance cassette and downstream of target gene. Briefly, the ∼620 bp upstream sequence and ∼620 bp downstream sequence of *C237_RS0105470* were amplified from the genome of *R. columbina* using the primers RC-Up P1 and RC-Up P2, RC-Down P1 and RC-Down P2, respectively. The ∼620 bp upstream sequence and ∼620 bp downstream sequence of *gldH* were amplified from the genome of *F. johnsoniae* using the primers Up(gldH) P1 and Up(gldH) P2, Down(gldH) P1 and Down(gldH) P2, respectively. An erythromycin resistance cassette was amplified from the genome of *R. anatipestifer* CH-1 using the primers RC-Erm P1 and RC-Erm P2 or Erm(gldH) P1 and Erm(gldH) P2, respectively (Liao et al., 2015; Luo et al., 2015). The three fragments were fused using overlapping PCR (Xiong et al., 2006; Huang et al., 2017, 2019). The fused fragments served as donor DNA for natural transformation.

### Natural Transformation Procedure

The procedure of natural transformation was similar to that used for *R. anatipestifer* described in a previous study (Liu et al., 2017; Huang et al., 2019). Briefly, *R. columbina* and *F. johnsoniae* were cultured in GCB liquid with shaking at 37°C for *R. columbina* and at 25°C for *F. johnsoniae*. The bacteria were collected during the logarithmic phase (OD_600_ = 3–4 for *R. columbina*, OD_600_ = 1–1.5 for *F. johnsoniae*) and adjusted to an optical density (OD) of 1. The growth curve of *F. johnsoniae* in GCB was shown in Supplementary Figure 1. The donor DNA was added to the bacterial cells and incubated for 1 h at 37°C for *R. columbina* and at 25°C for *F. johnsoniae*. Then, 100 μl of cells were plated on GCB agar plates supplemented with erythromycin (1 μg/ml for *R. columbina*; 50 μg/ml for *F. johnsoniae*) to count transformants. Then, 10 μl of cells were serially diluted with PBS and plated on GCB agar plates to count viable bacteria. The transformation frequency (TF) was calculated as transformants divided by viable bacteria. Then, 100 μl of cells were plated on GCB supplemented with the corresponding concentration of erythromycin to check for spontaneous mutants.

### Determination of Growth Curves

The bacteria were streaked on blood plates or GCB agar plates. A single colony was cultured in 5 ml of GCB liquid medium with shaking at 37°C for 14 h. The bacterial cells were transferred into 20 ml of GCB with or without peptone, phosphate or iron at an OD of 0.05 and cultured at 37°C with shaking. The OD_600_ was determined every 2 h for 14 h, and natural transformation was performed at the corresponding times.

### The Effect of Components of GCB on Natural Transformation in *R. columbina*

Bacterial cells were cultured to the logarithmic phase (OD_600_ = 3–4) and adjusted to an OD_600_ of 1. The bacterial cells were collected and resuspended in GCB medium depleted of vitamin B1 (VB_1_), glucose, L-glutamine, NaCl, peptone, or phosphate. After the bacteria were incubated at 37°C for 30 min, donor DNA was added to the cultured cells, and natural transformation was performed. Iron is essential for the growth of most bacteria (Liao et al., 2016). To investigate whether iron affects the growth and natural transformation of *R. columbina*, the growth curve of *R. columbina* in GCB supplemented with different concentrations of iron chelator ethylenediamine-N,N’-bis(2-hydroxyphenylacetic acid) (EDDHA) according to the method mentioned previously (Press et al., 2001; Liu et al., 2016, 2019) and natural transformation were performed after the bacteria were incubated into GCB supplemented with the corresponding concentration of EDDHA at 37°C for 30 min. The viable bacteria and transformants were counted, and the TF was calculated.

### EDTA Treatment

Bacterial cells were cultured until the logarithmic phase (OD600 = 3–4) at 37°C with shaking and adjusted to an OD_600_ of 1. Three hundred microliters of bacteria were collected and resuspended in GCB medium supplemented with 0.5 mM EDTA. Natural transformation was performed after the bacteria were incubated at 37°C for 30 min. The TF was calculated according to the method described previously. To investigate which divalent cation affects the natural transformation of *R. columbina*, different concentrations of CaCl_2_, MgCl_2_, ZnCl_2_, MnCl_2_, or CuCl_2_ were added into the GCB medium supplemented with the corresponding concentration of EDTA. The bacterial cells were first incubated in the above medium at 37°C for 30 min, and natural transformation was then performed. The TF was calculated as described previously.

### Statistics

Statistical analysis was performed using GraphPad Prism 8.0 (GraphPad Software Inc., La Jolla, United States). An unpaired two-tailed Student’s *t*-test was used to compare two groups, and a value of *P* < 0.05 was considered significant. Data represent the mean and standard deviation (SD) from at least three independent experiments.

## Results

### *R. columbina*, but Not *F. johnsoniae*, Is Naturally Competent Under the Same Conditions

**FIGURE 1:**
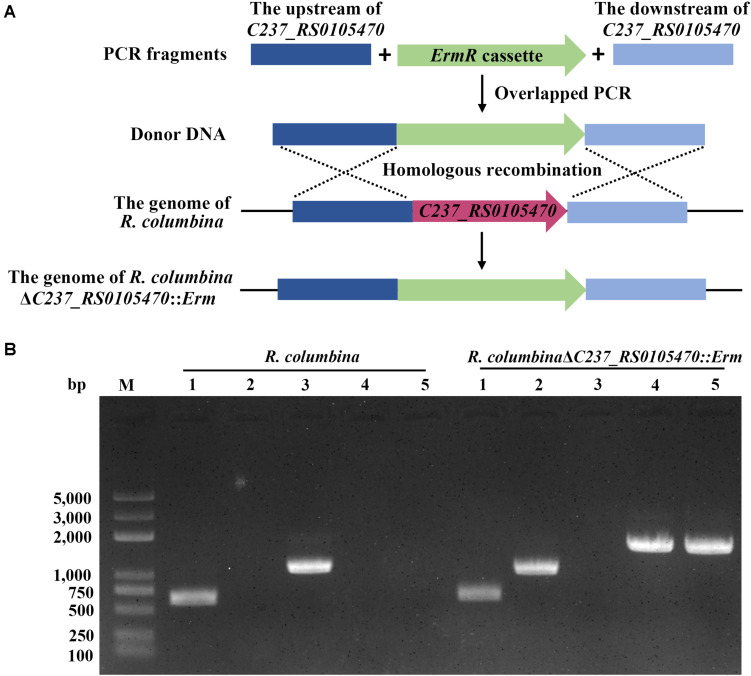
Verification of the mutant strain *R. columbina*Δ*C237_RS0105470*::*Erm*. **(A)** Schematic diagram of donor DNA preparation and gene deletion based on natural transformation. **(B)** The identification of the *R. columbina*Δ*C237_RS0105470:Erm* mutant strain by PCR. Lane M, MD103 DNA marker (BioMed, Beijing, China). Lane 1, the 16S rRNA was amplified from wild-type *R. columbina* and the mutant strain *R. columbina*Δ*C237_RS0105470*::*Erm* using the primers RC-16S rRNA P1 and RC-16S rRNA P2. Lane 2, the erythromycin resistance cassette was amplified from wild-type *R. columbina* and the mutant strain *R. columbina*Δ*C237_RS0105470*::*Erm* using the primers RC-Erm P1 and RC-Erm P2. Lane 3, the target gene *C237_RS0105470* was amplified from wild-type *R. columbina* and the mutant strain *R. columbina*Δ*C237_RS0105470*::*Erm* using the primers C237_RS0105470 P1 and C237_RS0105470 P2. Lane 4, the upstream sequence and erythromycin resistance cassette was amplified from wild-type *R. columbina* and the mutant strain *R. columbina*Δ*C237_RS0105470*::*Erm* using the primers RC-Up P1 and RC-Erm P2. Lane 5, the erythromycin resistance cassette and downstream sequence was amplified from wild-type *R. columbina* and the mutant strain *R. columbina*Δ*C237_RS0105470*::*Erm* using the primers RC-Erm P1 and RC-Down P2.

To assay whether other members of *Flavobacteriaceae* were able to undergo natural transformation, *R. columbina* and *F. johnsoniae* were selected. We used the same method as described in a previous study for *R. anatipestifer* to determine the natural competence of these two species (Liu et al., 2017). After *R. columbina* incubated with donor DNA which contains the upstream sequence of *C237_RS0105470*, an erythromycin resistance cassette and the downstream sequence of *C237_RS0105470* (Figure 1A), many resistant colonies grew on the plate with erythromycin. However, no resistant colonies appeared in the control group without donor DNA (the spontaneous mutation rate of erythromycin resistance was lower than the detection limitation). Random single colonies were verified using PCR to ensure that the target gene was replaced by the erythromycin resistance cassette through homologous recombination. As shown in Figure 1B, compared to the wild-type strain, the resistant colonies contained an erythromycin resistance gene but not a target gene. It was suggested that the target gene has been replaced by the erythromycin resistance cassette and that the target sequence of the *R. columbina* strain was lost. It was strongly supported that *R. columbina* was naturally competent and that natural transformation could be used to efficiently generate targeted gene disruptions in *R. columbina*, with a TF of 4.14 (±0.5) × 10^–6^.

After *F. johnsoniae* incubated with donor DNA containing the upstream sequence of *gldH*, an erythromycin resistance cassette and the downstream sequence of *gldH*, the transformants were selected on GCB plates supplemented with 50 μg/ml erythromycin (the MIC of erythromycin for *F. johnsoniae* is 16 μg/ml). However, no resistant colony appeared with or without donor DNA. It has been shown that *gldH* can be deleted in *F. johnsoniae* through other methods (McBride et al., 2003), suggesting that this gene is not essential for the survival of the bacteria. Overall, it was suggested that *F. johnsoniae* could not perform natural transformation using the same method as *R. columbina* under the same conditions.

### Searching for the Components of the Natural Transformation Machinery in the Genome of *F. johnsoniae*

To investigate whether *F. johnsoniae* contains the homologous proteins that involved in natural transformation. We aligned the amino acids sequences of T4SS from *H. pylori* and *Agrobacterium tumefaciens*, T4P from *V. cholerae*, T2SS from *C. jejuni*, and other hypothetical competence proteins from *R. anatipestifer* with genome of *F. johnsoniae*. As shown in Table 2, only the homolog of ComB11 in T4SS, which is a putative VirB11-homologous ATPase (Karnholz et al., 2006), was found in *F. johnsoniae* and showed 40.8% identity with the ComB11 of *H. pylori*. Based on the T4P of *V. cholerae* (Seitz and Blokesch, 2013b), only the homologs of PilB, PilC, PilF, PilQ and PilT were discovered in *F. johnsoniae* and shared 48.41, 29.62, 25.98, 27.43, and 43.33% with each relative protein of *V. cholerae*, respectively. PilB and PilT are polymerization and depolymerization ATPases, respectively (Seitz and Blokesch, 2013b). PilC was an inner membrane platform protein which interacts with PilB and PilT to control both pilus assembly and disassembly (Takhar et al., 2013). PilF is pilolin protein which is essential for pilus biogenesis (Matthey and Blokesch, 2016). PilQ is a secretion pore, which plays a role in translocating pilus on the cell surface (Wolfgang et al., 2000). Furthermore, only the homologs of CtsD and CtsF were found in *F. johnsoniae* based on the T2SS of *C. jejuni* (Wiesner et al., 2003). CtsD is an outer membrane protein which has homology to the PilQ protein (Wiesner et al., 2003). CtsF is an inner membrane protein and shares similarity to PilG of *N. gonorrhoeae* which has homology to the PilC of *V. cholerae* (Tønjum et al., 1995). Other hypothetical competence protein of *R. anatipestifer*, like DprA, ComEC, RecA, Ssb, ComM and RadC is also present in *F. johnsoniae* (Liu et al., 2017). These results indicated that these homologs of *F. johnsoniae* may be sufficient to encode a T4P-type DNA uptake system in addition to the proteins usually needed for DNA translocation and cytoplasmic processing.

**TABLE 2:** Homologs of T4SS, T4P, T2SS, and other competence proteins in *F. johnsoniae.*

**T4SS**	**Protein ID**	**Homologs^*a*^**	**Identity^*b*^**
VirB1	AAZ50518.1	None	None
ComB2	HP_0015	None	None
ComB3	HP_0016	None	None
ComB4	HP_0017	None	None
VirB5	AAZ50522.1	None	None
ComB6	HP_0037	None	None
VirB7	AAZ50524.1	None	None
ComB8	HP_0038	None	None
ComB9	HP_0039/40	None	None
ComB10	HP_0041/42	None	None
ComB11	HP_1421	WP_012022707.1	40.80%
VirD4	HP_0524	None	None

**T4P**	**Protein ID**	**Homologs^*a*^**	**Identity^*b*^**

PilA	VC_2423	None	None
PilB	VC_2424	WP_012022707.1	48.41%
PilE	VC_0857	None	None
FimT	VC_0858	None	None
VC_0859	VC_0859	None	None
VC_0860	VC_0860	None	None
PilV	VC_0861	None	None
PilF	VC_1612	WP_012024651.1	25.98%
PilQ	VC_2630	WP_012022708.1	27.43%
PilP	VC_2631	None	None
PilO	VC_2632	None	None
PilN	VC_2633	None	None
PilM	VC_2634	None	None
PilC	VC_2425	WP_012022704.1	29.62%
PilT	VC_0462	WP_012022707.1	43.33%

**T2SS**	**Protein ID**	**Homologs^*a*^**	**Identity^*b*^**

CtsD	Cj1474c	WP_012022708.1	23.51%
CtsF	AAP87276.1	WP_012022704	24.21%
CtsP	Cj1473c	None	None
CtsR	Cj1475c	None	None
CtsW	Cj1028c	None	None
CtsG	Cj1343c	None	None
CtsE	Cj1471c	None	None

**Others**	**Protein ID**	**Homologs^*a*^**	**Identity^*b*^**

DprA	RA0C_RS05130	WP_012023081.1	37.91%
ComEC	RA0C_RS04895	WP_012023505.1	24.45%
RecA	RA0C_RS04870	WP_012023074.1	77.08%
ComM	RA0C_RS07335	WP_012024210.1	74.56%
Ssb	RA0C_RS02530	WP_012022955.1	65.71%
RadC	RA0C_RS03540	WP_012022505.1	56.89%

*^*a*^Homologs in *F. johnsoniae*.*

*^b^Amino acids identity between *F. johnsoniae* and the example in the table.*

### Natural Transformation of *R. columbina* Increases During the Logarithmic Phase

**FIGURE 2:**
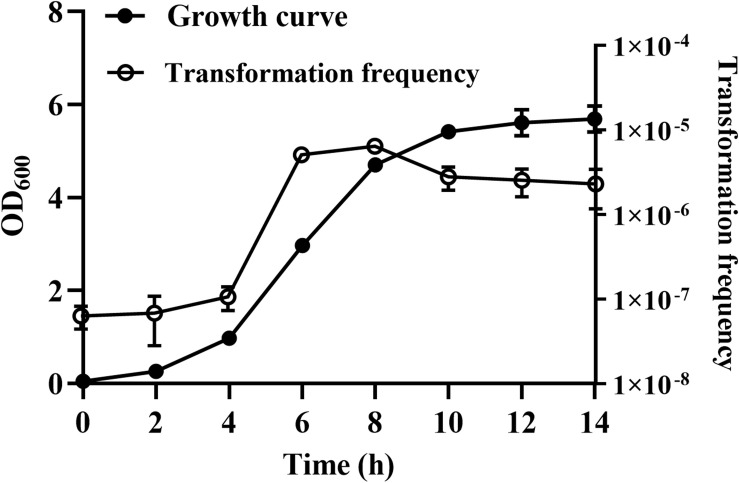
The effect of different growth phases on natural transformation. *R. columbina* was cultured with shaking at an OD_600_ of 0.05 for 14 h. The OD_600_ value was determined every 2 h. Additionally, natural transformation was assessed every 2 h. The results are representative of three independent experiments. Error bars denote standard deviation.

**FIGURE 3:**
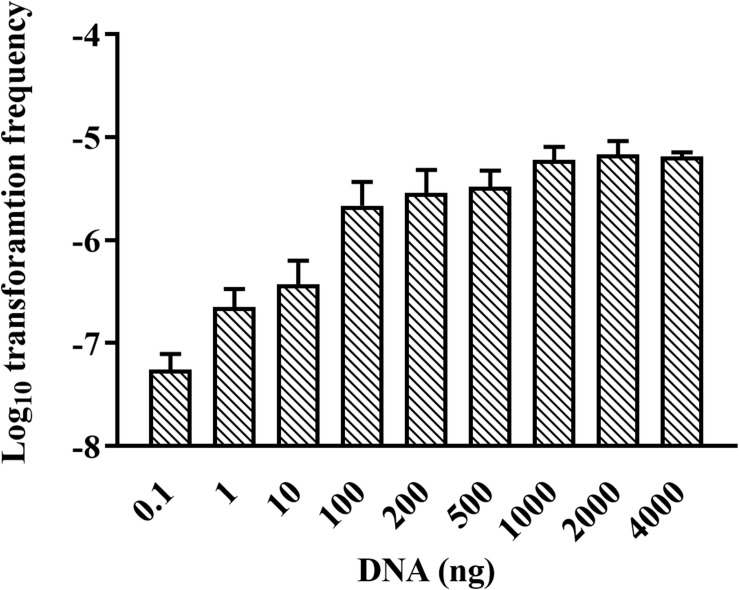
Effect of donor DNA amount on the TF of *R. columbina*. Donor DNA (0.1 to 4,000 ng) was added to 300 μl of cell cultures at an OD_600_ of 1 and incubated at 37°C for 1 h. The viable bacteria and erythromycin-resistant transformants were counted. The TF was calculated as transformants divided by the number of viable bacteria. All the results are representative of three independent experiments. Error bars denote standard deviation.

We were wondering whether natural transformation was able to occur in all growth phases in *R. columbina*, the TF was assayed. Natural transformation was performed at each time point by adding the same amount of donor DNA. As shown in Figure 2, natural formation of *R. columbina* occurred in all growth phases, and the TF was the highest during the logarithmic phase [TF = 6.45 (±0.55) × 10^–6^] and lowest in the lag phase [TF = 6.35 (±0.5) × 10^–8^]. The number of transformants in different growth phases were included in the Supplementary Data Sheet 2. To investigate the saturated concentration of donor DNA for logarithmic growth period bacteria, *R. columbina* was cultured to the logarithmic phase and mixed with different amounts of donor DNA (0.1, 1, 10, 100, 200, 500, 1,000, 2,000, or 4,000 ng). As shown in Figure 3, the TF increased with increasing DNA concentration when the amount was lower than 1,000 ng. However, when the DNA amount was higher than 1,000 ng, the TF no longer increased. The number of transformants were included in the Supplementary Data Sheet 2. These results suggested that 1,000 ng of donor DNA was saturating for natural transformation in *R. columbina*.

### *R. columbina* Preferentially Takes Up Its Own DNA Over Heterologous DNA

**FIGURE 4:**
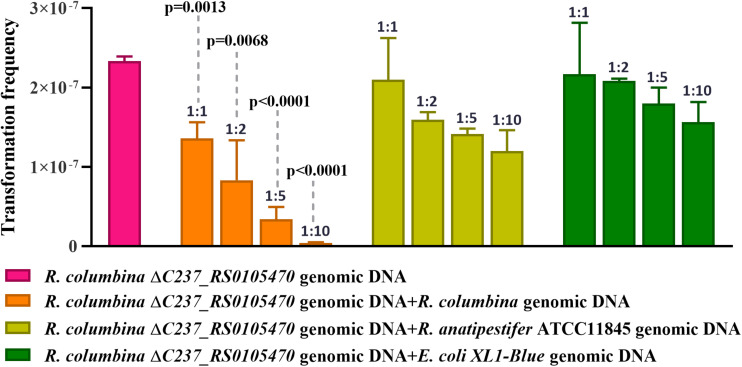
Natural transformation competition experiments. *R. columbina* was cultured to the logarithmic phase (3–4), and the OD_600_ value was determined. The cell culture was adjusted to an OD_600_ of 1. A 300 μl cell culture was transformed with 1 μg the genome of *R. columbina ΔC237_RS0105470* (control) or 1 μg of donor DNA mixed with different quantity of competing genomic DNA of *R. columbina*, *R. anatipestifer* ATCC11845 or *E. coli* XL1-Blue and incubated at 37°C for 1 h. The natural TF was calculated as transformants divided by viable bacteria. The *P*-value refers to the difference between the no-competition and competition measurement, respectively. Insignificance *p*-value (*P* > 0.05) was not shown. All the results are representative of three independent experiments. Error bars denote standard deviation.

It has been reported that some bacteria, such as *H. influenzae* and *Neisseria*, preferentially take up DNA containing short motifs known as uptake signal sequences (USSs) or DNA uptake sequences (DUSs) (Scocca et al., 1974; Sisco and Smith, 1979). These short motifs have accumulated in the genome to high densities over evolutionary time (Mell and Redfield, 2014). To determine whether *R. columbina* also preferentially takes up its own DNA, a natural transformation competition experiment was performed. In this experiment, the genome of *R. columbina ΔC237_RS0105470* was used as the donor DNA. As shown in Figure 4, when 1 μg of donor DNA was mixed with 1 μg of genomic DNA of *R. columbina*, the TF was decreased two-fold compared to the control in which without competition DNA was added; Moreover, the TF was decreased with the increase of competing DNA. However, the TF showed no significant changes when 1 μg donor of DNA was mixed with 1 μg of *R. anatipestifer* or *E. coli* genomic DNA compared to that when only 1 μg of donor DNA was added. The TF was decreased significantly only when the *R. anatipestifer* or *E. coli* genomic DNA was increased to 10 μg, which can be considered as unspecific effect. The number of transformants were included in the Supplementary Data Sheet 2. To further investigate whether *R. columbina*, *F. johnsoniae* or *R. anatipestifer* contain putative DUSs or USSs, Jellyfish^1^ was to be used to count the numbers of occurrences of individual kmers in both strands of their genome, respectively, with a parameter that limited the length of kmers to less than 10 bp. As shown in Table 3, sequences with the top three repeats for 10 bp, 9 bp, 8 bp, and 7 bp were listed, respectively. It was found that hundreds of repeat sequences or its complement were present in their genomes. The frequency of the 9-bp repeat sequence is 0.6/kb for *R. anatipestifer*, 0.5/kb for *R. columbina* and 0.5/kb for *F. johnsoniae*, respectively, which is much higher than the frequency of 0.1/kb expected for a random sequence of this base composition for them. Whether this sequence has the function of DUSs or USSs needs to be further investigated.

**TABLE 3:** Analysis of putative DUSs or USSs in *R. anatipestifer*, *R. columbina* and *F. johnsoniae.*

**kmer**	***R. anatipestifer***			***R. columbina***			***F. johnsoniae***		
	**Sequence**	**Repeats**	**Expected repeats^*a*^**	**Sequence**	**Repeats**	**Expected repeats^*a*^**	**Sequence**	**Repeats**	**Expected repeats^*a*^**
10	AAAAATAAAA	301	56	AAAAAGAAAA	203	30	AAAAAATAAA	759	183
	AAAAAATAAA	235	56	AAAAAAATAA	196	54	AAAAACAAAA	743	95
	AAAAAAATAA	201	56	AAAATTAAAA	196	54	AAAAAAATAT	479	183
9	AAAATAAAA	650	175	AAAAATAAA	666	171	TTTTAAAAA	1769	558
	AAAAAATAA	534	175	AAAAAATAA	551	171	AAAAAATAA	1591	558
	AAAAAAATA	520	175	AAAAAAATA	514	171	AAAAACAAA	1569	288
8	AAAAATAA	1428	538	AAAAAATA	1534	535	TTTAAAAA	4593	1694
	AAAATAAA	1424	538	AAAAATAA	1522	535	AAAAAATA	4095	1694
	AAAAAATA	1412	538	AAAATAAA	1510	535	AAAAAAAT	3910	1694
7	AAAAATA	3872	1657	AAAAATA	4210	1674	TTTAAAA	13724	5141
	AAAATAA	3185	1657	AAAAAAT	4114	1674	AAAAAAA	11435	5141
	AAAAAAA	2881	1657	AAAATAA	3445	1674	AAAAAAT	11213	5141

*^*a*^Expected repeats is calculated directly from the base composition and length of the genome.*R. columbina* (2436790 bp) and *F. johnsoniae* (6096872 bp) and the GC content of each strain: *R. anatipestifer* (35%), *R. columbina* (36%) and *F. johnsoniae* (34.1%).*

### The TF of *R. columbina* Is Increased Under Peptone-Restrictive or Phosphate-Restrictive Conditions

**FIGURE 5:**
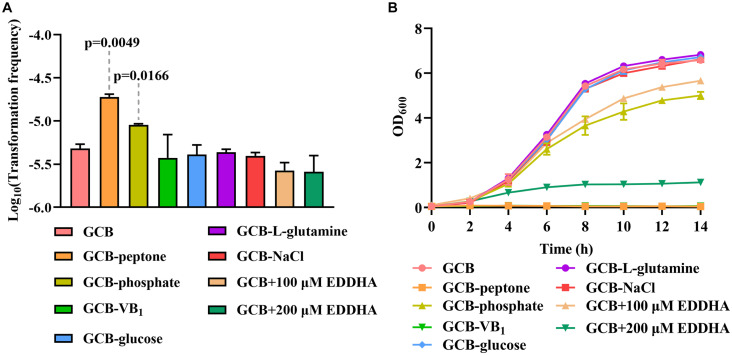
The effect of GCB ingredients on natural transformation and growth in *R. columbina*. **(A)** TFs of *R. columbina* in GCB, GCB depleted of VB_1_, glucose, L-glutamine, NaCl, peptone or phosphate and GCB supplemented with different concentrations of EDDHA. The *P*-value refers to the difference between the TF in GCB and in GCB depleted of peptone or phosphate, respectively. Insignificance *p*-value (*P* > 0.05) was not shown. **(B)** Growth curves of *R. columbina* in GCB, GCB depleted of VB_1_, glucose, L-glutamine, NaCl, peptone, or phosphate and GCB supplemented with different concentrations of EDDHA. The bacteria were cultured in 20 ml of the above medium inoculated at an OD_600_ of 0.05, and the OD_600_ value was determined every 2 h. All the results are representative of three independent experiments. Error bars denote standard deviation.

To investigate the effect of the nutrients on natural transformation, natural transformation was conducted in GCB depleted for each component, including vitamin B1 (VB_1_), glucose, L-glutamine, NaCl, peptone and phosphate. As shown in Figure 5A, the TF of *R. columbina* was 1.9 (±0.1) × 10^–5^ in GCB depleted of peptone, which increased five-fold compared to that in GCB. The TF of *R. columbina* was 9.05 (±0.5) × 10^–6^ in GCB depleted of phosphate, which increased approximately two-fold compared to that in GCB. However, compared to the TF of *R. columbina* in GCB, there was no significant difference when VB_1_, glucose, L-glutamine or NaCl was removed from GCB (Figure 5A). The number of transformants were included in the Supplementary Data Sheet 2.

Next, we investigated whether the change in TF was associated with the growth ability of bacteria. Therefore, the growth curve of bacteria was determined when peptone, phosphate, NaCl, glucose, L-glutamine or VB_1_ was removed from GCB. The results showed that bacteria did not grow in GCB without peptone or VB_1_, whereas the growth of bacteria was significantly decreased in GCB without phosphate; however, there were no significant differences when NaCl, glucose, or L-glutamine was removed from GCB (Figure 5B). To investigate whether iron affects the natural transformation of *R. columbina*, different concentrations of iron chelator EDDHA were supplemented into the GCB medium. As shown in Figure 5A, the TF did not change compared to that of the control (without EDDHA). The number of transformants were included in the Supplementary Data Sheet 2. However, the growth of *R. columbina* was significantly inhibited in iron-depleted medium (GCB supplemented with 200 μM EDDHA), suggesting that iron is essential for the growth of *R. columbina* (Figure 5B). Overall, these results suggested that peptone-restrictive or phosphate-restrictive medium had an effect on the natural transformation and this effect is not directly related to the growth ability.

### Ca^2+^, Mg^2+^, Zn^2+^, and Mn^2+^ but Not Cu^2+^ Promote the Natural Transformation of *R. columbina*

**FIGURE 6:**
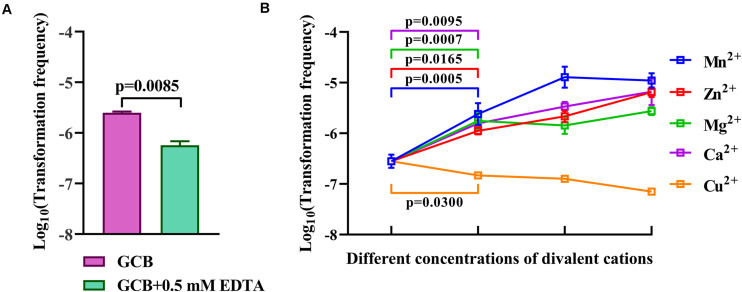
Effect of EDTA and different divalent cations on the natural transformation of *R. columbina*. **(A)** Viable bacteria were counted after treatment with or without 0.5 mM EDTA for 1 h, and the effect of 0.5 mM EDTA on the natural transformation of *R. columbina* was determined. The *P*-value refers to the difference between the TF in GCB and in GCB containing 0.5 mM EDTA. **(B)** The natural TF of *R. columbina* in GCB supplemented with 0.5 mM EDTA and different supplemented concentrations of divalent cation. The concentration of Ca^2+^ are 0.5, 1, and 5 mM, respectively. The concentration of Mg^2+^ are 0.5, 1, and 5 mM, respectively. The concentration of Zn^2+^ are 0.1, 0.2, and 0.5 mM, respectively. The concentration of Mn*^2+^* are 0.2, 0.5, and 1 mM, respectively. The concentration of Cu^2+^ are 0.1, 0.2, and 0.5 mM, respectively. The *P*-value refers to the difference between the TF in GCB containing 0.5 mM EDTA and GCB containing 0.5 mM EDTA supplemented by Ca^2+^, Mg^2+^, Mg^2+^, or Cu^2+^, respectively. All the results are representative of three independent experiments. Error bars denote standard deviation.

Iron has no effect on natural transformation, and we wondered if other divalent cations influence the natural transformation of *R. columbina*. Natural transformation was conducted in GCB medium with 0.5 mM EDTA, which had no effect on the survival of bacteria (Supplementary Figure 2). The results showed that the TF in GCB with 0.5 mM EDTA was 5.75 (±0.75) × 10^–7^, which was decreased approximately 4-fold compared to that in GCB [TF = 2.5(±0.1) × 10^–6^], suggesting that 0.5 mM EDTA had a significant inhibitory effect on natural transformation in *R. columbina* (Figure 6A). To investigate which divalent cation has an effect on natural transformation, different concentrations of CaCl_2_, MgCl_2_, ZnCl_2_, MnCl_2_, or CuCl_2_ were supplemented into the cell culture after incubation with EDTA. The addition of 0.5 mM Ca^2+^ basically restored transformation, and the TF increased as the concentration of Ca^2+^ increased (Figure 6B). The addition of 0.5 mM Mg^2+^ completely restored the TF; however, the frequency did not increase as the concentration of Mg^2+^ increased (Figure 6B), suggesting that 0.5 mM was likely a saturating concentration of Mg^2+^ for natural transformation in *R. columbina*. The TF gradually increased as the concentration of Zn^2+^ increased from 0.1 mM to 0.5 mM. The TF was the highest at 0.5 mM Mn^2+^(Figure 6B). However, the addition of different concentrations of Cu^2+^ did not restore the natural transformation but instead inhibited natural transformation (Figure 6B). The number of transformants were included in the Supplementary Data Sheet 2. Therefore, it was shown that Ca^2+^, Mg^2+^, Zn^2+^, and Mn^2+^ were required for the natural transformation of *R. columbina*, but Cu^2+^ was not.

## Discussion

*Riemerella anatipestifer* is the first bacterium of *Flavobacteriaceae* to be reported to have natural competence (Liu et al., 2017). To check whether other bacteria in *Flavobacteriaceae* are also naturally competent, *F. johnsoniae* and *R. columbina* were selected. The results showed that *R. columbina* was able to undergo natural transformation; however, *F. johnsoniae* was not competent under the same conditions. One possibility is that the natural transformation of *F. johnsoniae* does not occur at all growth phases but only at a certain time point, for example natural transformation happens to *S. pneumoniae*, in which natural transformation is not constitutive, as synthesis and assembly of the uptake apparatus is a transient and regulated process (Mirouze et al., 2013). Another possibility is that the PCR fragments are not suitable substrates, such as occurs with *C. jejuni*, which takes up only methylated DNA but not PCR fragments (Beauchamp et al., 2017), and *H. influenzae* and *Neisseria gonorrhoeae*, which preferentially take up DNA with an USS or DUS over other sources of DNA (Mell et al., 2012; Berry et al., 2013; Frye et al., 2013; Mell and Redfield, 2014). The third possibility is that natural transformation in *F. johnsoniae* must be induced by special substrates, such as occurs with natural transformation of *Vibrio cholerae*, which is induced by chitin (Meibom et al., 2005). The fourth possibility is that we did not choose the correct isolates. It has been reported that even for the competent bacteria, some isolates are non-transformable (Evans and Rozen, 2013; Dalia et al., 2015). The last possibility is that *F. johnsoniae* does not undergo natural transformation because of the lack of some essential genes for natural transformation.

Consistent with the natural transformation of *R. anatipestifer* (Liu et al., 2017), the natural transformation of *R. columbina* is also constitutive, although the TF is different at the different growth phases. This phenomenon might occur because the expression of genes involved in natural transformation in *R. columbina* is different at the different growth phases. Similar to *R. anatipestifer*, *R. columbina* preferentially takes up self-sourced genomic DNA, suggesting that each bacterium might use a certain mechanism, such as a restriction modification (R-M) system (Aras et al., 2002; Zhang and Blaser, 2012) or other systems, to prevent the uptake of excessive extracellular DNA that may overload the bacteria, subverting the bacterial genome with extracellular DNA from competing strains.

Originally, the function of natural transformation was hypothesized as “DNA for food” (Redfield, 2001), because the natural competence of *H. influenzae* and *B. subtilis* was activated under nutrient-limited condition (Bobb, 1963; Herriott et al., 1970). However, this hypothesis is questionable, since the natural competence of some other bacteria, such as *A. baumannii*, requires a nutrient-rich condition (Traglia et al., 2016). In the case of *R. columbina*, we showed that the TF of *R. columbina* was significantly increased under peptone-restrictive or phosphate-restrictive conditions, suggesting that the uptake of DNA may be “food” for *R. columbina* to supplement the nitrogen and phosphorus.

A more plausible hypothesis for the function of natural transformation is “DNA for repair”(Michod et al., 2008; Engelmoer et al., 2013), since the natural transformation of some bacteria, such as *H. pylori* (Dorer et al., 2010), *S. pneumoniae* (Prudhomme et al., 2006) and *B. subtilis* (Zhang et al., 2018), was activated by antibiotics or DNA damage reagent. Here, we also investigated the effects of antibiotics such as ampicillin (inhibitor of cell wall biosynthesis), kanamycin (inhibitor of protein biosynthesis), nalidixic acid (inhibitor of DNA replication) and mitomycin C (intercalation with DNA) during the natural transformation of *R. columbina*. We showed that none of the antibiotics affected natural TF of *R. columbina* did not change after treatment with antibiotics (Supplementary Figure 3), suggesting that the antibiotics used here cannot trigger natural transformation of *R. columbina*.

Our systematic investigation of natural transformation in the *Flavobacteriaceae* family shows that it is widely distributed. However, the environmental conditions that trigger natural transformation vary from species to species. In this family, natural transformation appears to play a major role in HGT. The discovery of natural transformation in *R. columbina* represents the basis for the establishment of gene editing and cloning system in this bacterium.

## Data Availability Statement

The original contributions presented in the study are included in the article/Supplementary Material, further inquiries can be directed to the corresponding author/s.

## Author Contributions

ML, DZ, and AC conceived and designed the experiments. LH, LX, MH, CX, SZ, QG, DS, and BT performed the experiments. MW, RJ, SC, XZ, QY, and YW analyzed the data. JH, XO, and SM contributed to reagents, materials, and analysis tools. ML, FB, and AC wrote the manuscript. All authors have reviewed the manuscript.

## Conflict of Interest

The authors declare that the research was conducted in the absence of any commercial or financial relationships that could be construed as a potential conflict of interest.

## Footnotes

1 https://github.com/gmarcais/Jellyfish

## References

[B1] Aras R. A. Small A. J. Ando T. Blaser M. J. (2002). *Helicobacter pylori* interstrain restriction-modification diversity prevents genome subversion by chromosomal DNA from competing strains. *Nucleic Acids Res.* 30 5391–5397. 10.1093/nar/gkf686 12490707 PMC140068

[B2] Beauchamp J. M. Leveque R. M. Dawid S. DiRita V. J. (2017). Methylation-dependent DNA discrimination in natural transformation of *Campylobacter jejuni*. *Proc. Natl. Acad. Sci. U.S.A.* 114 E8053–E8061. 10.1073/pnas.1703331114 28855338 PMC5617262

[B3] Berry J. L. Cehovin A. McDowell M. A. Lea S. M. Pelicic V. (2013). Functional analysis of the interdependence between DNA uptake sequence and its cognate ComP receptor during natural transformation in Neisseria species. *PLoS Genet.* 9:e1004014. 10.1371/journal.pgen.1004014 24385921 PMC3868556

[B4] Blokesch M. (2012). Chitin colonization, chitin degradation and chitin-induced natural competence of *Vibrio cholerae* are subject to catabolite repression. *Environ. Microbiol.* 14 1898–1912. 10.1111/j.1462-2920.2011.02689.x 22222000

[B5] Bobb D. (1963). Overnight incubation technique for obtaining transformable *Bacillus subtilus* cells of reproducible competency. *Nature* 199 828–829. 10.1038/199828a0 14071214

[B6] Dalia A. B. Seed K. D. Calderwood S. B. Camilli A. (2015). A globally distributed mobile genetic element inhibits natural transformation of *Vibrio cholerae*. *Proc. Natl. Acad. Sci. U.S.A.* 112 10485–10490. 10.1073/pnas.1509097112 26240317 PMC4547284

[B7] Dalia T. N. Yoon S. H. Galli E. Barre F. X. Waters C. M. Dalia A. B. (2017). Enhancing multiplex genome editing by natural transformation (MuGENT) via inactivation of ssDNA exonucleases. *Nucleic Acids Res.* 45 7527–7537. 10.1093/nar/gkx496 28575400 PMC5499599

[B8] Dorer M. S. Fero J. Salama N. R. (2010). DNA damage triggers genetic exchange in *Helicobacter pylori*. *PLoS Pathog.* 6:e1001026. 10.1371/journal.ppat.1001026 20686662 PMC2912397

[B9] Engelmoer D. J. Donaldson I. Rozen D. E. (2013). Conservative sex and the benefits of transformation in *Streptococcus pneumoniae*. *PLoS Pathog.* 9:e1003758. 10.1371/journal.ppat.1003758 24244172 PMC3828180

[B10] Evans B. A. Rozen D. E. (2013). Significant variation in transformation frequency in *Streptococcus pneumoniae*. *ISME J.* 7 791–799. 10.1038/ismej.2012.170 23303370 PMC3603394

[B11] Frye S. A. Nilsen M. Tonjum T. Ambur O. H. (2013). Dialects of the DNA uptake sequence in Neisseriaceae. *PLoS Genet.* 9:e1003458. 10.1371/journal.pgen.1003458 23637627 PMC3630211

[B12] Griffith F. (1928). The significance of pneumococcal types. *J. Hyg.* 27 113–159. 10.1017/s0022172400031879 20474956 PMC2167760

[B13] Hahn J. Maier B. Haijema B. J. Sheetz M. Dubnau D. (2005). Transformation proteins and DNA uptake localize to the cell poles in Bacillus subtilis. *Cell* 122 59–71. 10.1016/j.cell.2005.04.035 16009133 PMC4442496

[B14] Herriott R. M. Meyer E. M. Vogt M. (1970). Defined nongrowth media for stage II development of competence in *Haemophilus influenzae*. *J. Bacteriol.* 101 517–524. 10.1128/jb.101.2.517-524.1970 5308771 PMC284936

[B15] Hofreuter D. Odenbreit S. Puls J. Schwan D. Haas R. (2000). Genetic competence in *Helicobacter pylori*: mechanisms and biological implications. *Res. Microbiol.* 151 487–491. 10.1016/s0923-2508(00)00164-9 10961464

[B16] Hovland E. Beyene G. T. Frye S. A. Homberset H. Balasingham S. V. Gomez-Munoz M. (2017). DprA from *Neisseria meningitidis*: properties and role in natural competence for transformation. *Microbiology* 163 1016–1029. 10.1099/mic.0.000489 28696187 PMC5817196

[B17] Huang L. Tian X. Liu M. Wang M. Biville F. Cheng A. (2019). DprA is essential for natural competence in riemerella anatipestifer and has a conserved evolutionary mechanism. *Front. Genet.* 10:429. 10.3389/fgene.2019.00429 31156696 PMC6533540

[B18] Huang L. Yuan H. Liu M. F. Zhao X. X. Wang M. S. Jia R. Y. (2017). Type B chloramphenicol acetyltransferases are responsible for chloramphenicol resistance in *Riemerella anatipestifer*, China. *Front. Microbiol.* 8:297. 10.3389/fmicb.2017.00297 28298905 PMC5331189

[B19] Johnston C. Martin B. Fichant G. Polard P. Claverys J. P. (2014). Bacterial transformation: distribution, shared mechanisms and divergent control. *Nat. Rev. Microbiol.* 12 181–196. 10.1038/nrmicro3199 24509783

[B20] Karnholz A. Hoefler C. Odenbreit S. Fischer W. Hofreuter D. Haas R. (2006). Functional and topological characterization of novel components of the comB DNA transformation competence system in *Helicobacter pylori*. *J. Bacteriol.* 188 882–893. 10.1128/jb.188.3.882-893.2006 16428391 PMC1347336

[B21] Li J. Yuan X. Xu L. Kang L. Jiang J. Wang Y. (2016). Efficient construction of *Haemophilus parasuis* mutants based on natural transformation. *Can. J. Vet. Res.* 80 281–286. 27733782 PMC5052879

[B22] Liao H. Cheng X. Zhu D. Wang M. Jia R. Chen S. (2015). TonB energy transduction systems of *Riemerella anatipestifer* are required for iron and hemin utilization. *PLoS One* 10:e0127506. 10.1371/journal.pone.0127506 26017672 PMC4446302

[B23] Liao H. Liu M. Cheng X. Zhu D. Wang M. Jia R. (2016). The detection of hemin-binding proteins in *Riemerella anatipestifer* CH-1. *Curr. Microbiol.* 72 152–158. 10.1007/s00284-015-0932-5 26542531

[B24] Liu M. Huang M. Huang L. Biville F. Zhu D. Wang M. (2019). New perspectives on *Galleria mellonella* larvae as a host model using Riemerella anatipestifer as a proof of concept. *Infect. Immun*. 87:e00072-19. 10.1128/IAI.00072-19 31160365 PMC6652747

[B25] Liu M. Wang M. Zhu D. Wang M. Jia R. Chen S. (2016). Investigation of TbfA in *Riemerella anatipestifer* using plasmid-based methods for gene over-expression and knockdown. *Sci. Rep.* 6:37159. 10.1038/srep37159 27845444 PMC5109031

[B26] Liu M. Zhang L. Huang L. Biville F. Zhu D. Wang M. (2017). Use of natural transformation to establish an easy knockout method in *Riemerella anatipestifer*. *Appl. Environ. Microbiol.* 83:e000127-17. 10.1128/AEM.00127-17 28258143 PMC5394337

[B27] Luo H. Liu M. Wang L. Zhou W. Wang M. Cheng A. (2015). Identification of ribosomal RNA methyltransferase gene ermF in Riemerella anatipestifer. *Avian Pathol.* 44 162–168. 10.1080/03079457.2015.1019828 25690020

[B28] Matthey N. Blokesch M. (2016). The DNA-Uptake process of naturally competent *Vibrio cholerae*. *Trends Microbiol.* 24 98–110. 10.1016/j.tim.2015.10.008 26614677

[B29] McBride M. J. (2004). *Cytophaga-flavobacterium* gliding motility. *J. Mol. Microbiol. Biotechnol.* 7 63–71. 10.1159/000077870 15170404

[B30] McBride M. J. Braun T. F. Brust J. L. (2003). *Flavobacterium johnsoniae* GldH is a lipoprotein that is required for gliding motility and chitin utilization. *J. Bacteriol.* 185 6648–6657. 10.1128/jb.185.22.6648-6657.2003 14594839 PMC262120

[B31] Meibom K. L. Blokesch M. Dolganov N. A. Wu C. Y. Schoolnik G. K. (2005). Chitin induces natural competence in *Vibrio cholerae*. *Science* 310 1824–1827. 10.1126/science.1120096 16357262

[B32] Mell J. C. Hall I. M. Redfield R. J. (2012). Defining the DNA uptake specificity of naturally competent *Haemophilus influenzae* cells. *Nucleic Acids Res.* 40 8536–8549. 10.1093/nar/gks640 22753031 PMC3458573

[B33] Mell J. C. Redfield R. J. (2014). Natural competence and the evolution of DNA uptake specificity. *J. Bacteriol.* 196 1471–1483. 10.1128/JB.01293-13 24488316 PMC3993363

[B34] Michod R. E. Bernstein H. Nedelcu A. M. (2008). Adaptive value of sex in microbial pathogens. *Infect. Genet. Evol.* 8 267–285. 10.1016/j.meegid.2008.01.002 18295550

[B35] Mirouze N. Berge M. A. Soulet A. L. Mortier-Barriere I. Quentin Y. Fichant G. (2013). Direct involvement of DprA, the transformation-dedicated RecA loader, in the shut-off of pneumococcal competence. *Proc. Natl. Acad. Sci. U.S.A.* 110 E1035–E1044. 10.1073/pnas.1219868110 23440217 PMC3600483

[B36] Press C. M. Loper J. E. Kloepper J. W. (2001). Role of iron in rhizobacteria-mediated induced systemic resistance of cucumber. *Phytopathology* 91 593–598. 10.1094/PHYTO.2001.91.6.593 18943949

[B37] Prudhomme M. Attaiech L. Sanchez G. Martin B. Claverys J. P. (2006). Antibiotic stress induces genetic transformability in the human pathogen *Streptococcus pneumoniae*. *Science* 313 89–92. 10.1126/science.1127912 16825569

[B38] Redfield R. J. (1991). sxy-1, a *Haemophilus influenzae* mutation causing greatly enhanced spontaneous competence. *J. Bacteriol.* 173 5612–5618. 10.1128/jb.173.18.5612-5618.1991 1653215 PMC208288

[B39] Redfield R. J. (2001). Do bacteria have sex? *Nat. Rev. Genet.* 2 634–639. 10.1038/35084593 11483988

[B40] Redfield R. J. Findlay W. A. Bosse J. Kroll J. S. Cameron A. D. Nash J. H. (2006). Evolution of competence and DNA uptake specificity in the Pasteurellaceae. *BMC Evol. Biol.* 6:82. 10.1186/1471-2148-6-82 17038178 PMC1626085

[B41] Rubbenstroth D. Ryll M. Hotzel H. Christensen H. Knobloch J. K. Rautenschlein S. (2013). Description of Riemerella columbipharyngis sp. nov., isolated from the pharynx of healthy domestic pigeons (*Columba livia* f. domestica), and emended descriptions of the genus Riemerella, *Riemerella anatipestifer* and *Riemerella columbina*. *Int. J. Syst. Evol. Microbiol.* 63(Pt 1), 280–287. 10.1099/ijs.0.036798-0 22427448

[B42] Scocca J. J. Poland R. L. Zoon K. C. (1974). Specificity in deoxyribonucleic acid uptake by transformable *Haemophilus influenzae*. *J. Bacteriol.* 118 369–373. 10.1128/jb.118.2.369-373.1974 4597440 PMC246767

[B43] Seitz P. Blokesch M. (2013a). Cues and regulatory pathways involved in natural competence and transformation in pathogenic and environmental Gram-negative bacteria. *FEMS Microbiol. Rev.* 37 336–363. 10.1111/j.1574-6976.2012.00353.x 22928673

[B44] Seitz P. Blokesch M. (2013b). DNA-uptake machinery of naturally competent *Vibrio cholerae*. *Proc. Natl. Acad. Sci. U.S.A.* 110 17987–17992. 10.1073/pnas.1315647110 24127573 PMC3816411

[B45] Sisco K. L. Smith H. O. (1979). Sequence-specific DNA uptake in *Haemophilus* transformation. *Proc. Natl. Acad. Sci. U.S.A.* 76 972–976. 10.1073/pnas.76.2.972 311478 PMC383110

[B46] Takhar H. K. Kemp K. Kim M. Howell P. L. Burrows L. L. (2013). The platform protein is essential for type IV pilus biogenesis. *J. Biol. Chem.* 288 9721–9728. 10.1074/jbc.m113.453506 23413032 PMC3617274

[B47] Tønjum T. Freitag N. E. Namork E. Koomey M. (1995). Identification and characterization of pilG, a highly conserved pilus−assembly gene in pathogenic Neisseria. *Mol. Microbiol.* 16 451–464. 10.1111/j.1365-2958.1995.tb02410.x 7565106

[B48] Traglia G. M. Quinn B. Schramm S. T. Soler-Bistue A. Ramirez M. S. (2016). Serum Albumin and Ca2+ are natural competence inducers in the human pathogen *Acinetobacter baumannii*. *Antimicrob. Agents Chemother.* 60 4920–4929. 10.1128/AAC.00529-16 27270286 PMC4958237

[B49] Wiedenbeck J. Cohan F. M. (2011). Origins of bacterial diversity through horizontal genetic transfer and adaptation to new ecological niches. *FEMS Microbiol. Rev.* 35 957–976. 10.1111/j.1574-6976.2011.00292.x 21711367

[B50] Wiesner R. S. Hendrixson D. R. DiRita V. J. (2003). Natural transformation of *Campylobacter jejuni* requires components of a type II secretion system. *J. Bacteriol.* 185 5408–5418. 10.1128/jb.185.18.5408-5418.2003 12949093 PMC193740

[B51] Wolfgang M. van Putten J. P. Hayes S. F. Dorward D. Koomey M. (2000). Components and dynamics of fiber formation define a ubiquitous biogenesis pathway for bacterial pili. *Embo J.* 19 6408–6418. 10.1093/emboj/19.23.6408 11101514 PMC305860

[B52] Xiong A. S. Yao Q. H. Peng R. H. Duan H. Li X. Fan H. Q. (2006). PCR-based accurate synthesis of long DNA sequences. *Nat. Protoc.* 1 791–797. 10.1038/nprot.2006.103 17406309

[B53] Zhang L. Li Y. Dai K. Wen X. Wu R. Huang X. (2015). Establishment of a successive markerless mutation system in *Haemophilus parasuis* through natural transformation. *PLoS One* 10:e0127393. 10.1371/journal.pone.0127393 25985077 PMC4436007

[B54] Zhang X. Jin T. Deng L. Wang C. Zhang Y. Chen X. (2018). Stress-induced, highly efficient, donor cell-dependent cell-to-cell natural transformation in *Bacillus subtilis*. *J. Bacteriol*. 200:e00267-18. 10.1128/JB.00267-18 29941421 PMC6088163

[B55] Zhang X. S. Blaser M. J. (2012). Natural transformation of an engineered *Helicobacter pylori* strain deficient in type II restriction endonucleases. *J. Bacteriol.* 194 3407–3416. 10.1128/JB.00113-12 22522893 PMC3434758

