# Supplementary material for: Natural Transformation of Riemerella columbina and Its Determinants
Source: Front Microbiol. 2021 Mar 3;12:634895. doi: 10.3389/fmicb.2021.634895 (PMC7965970; doi:10.3389/fmicb.2021.634895)
Supplement: Supplementary file 1 [file Data_Sheet_1.docx]

Supplementary Material


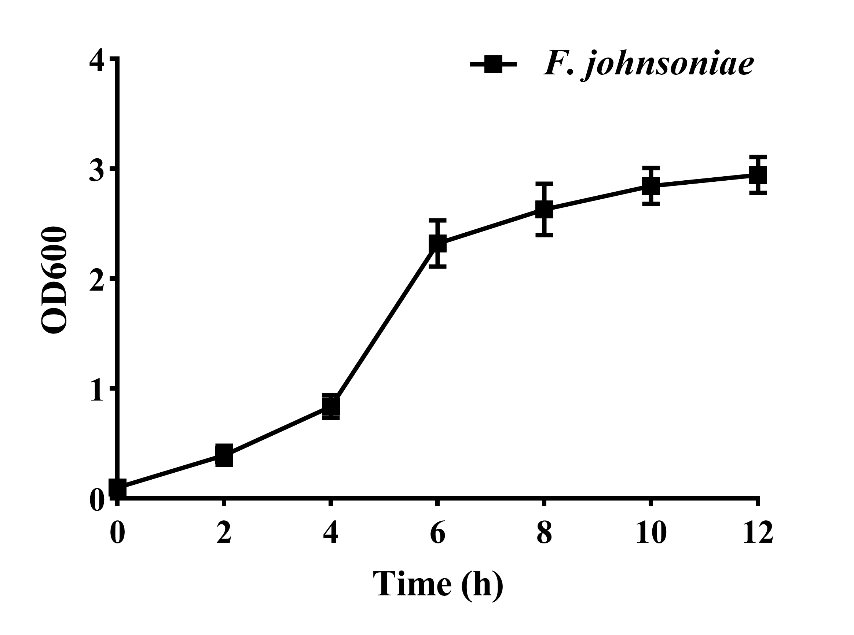


**Supplementary Figure 1 The growth curve of *F. johnsoniae* in GCB.** *F. johnsoniae* were cultured into the 20 ml GCB at the initial OD600 of 0.1 and incubated at 25°C with shaking. The OD600 was measured every 2 h for 12 h.


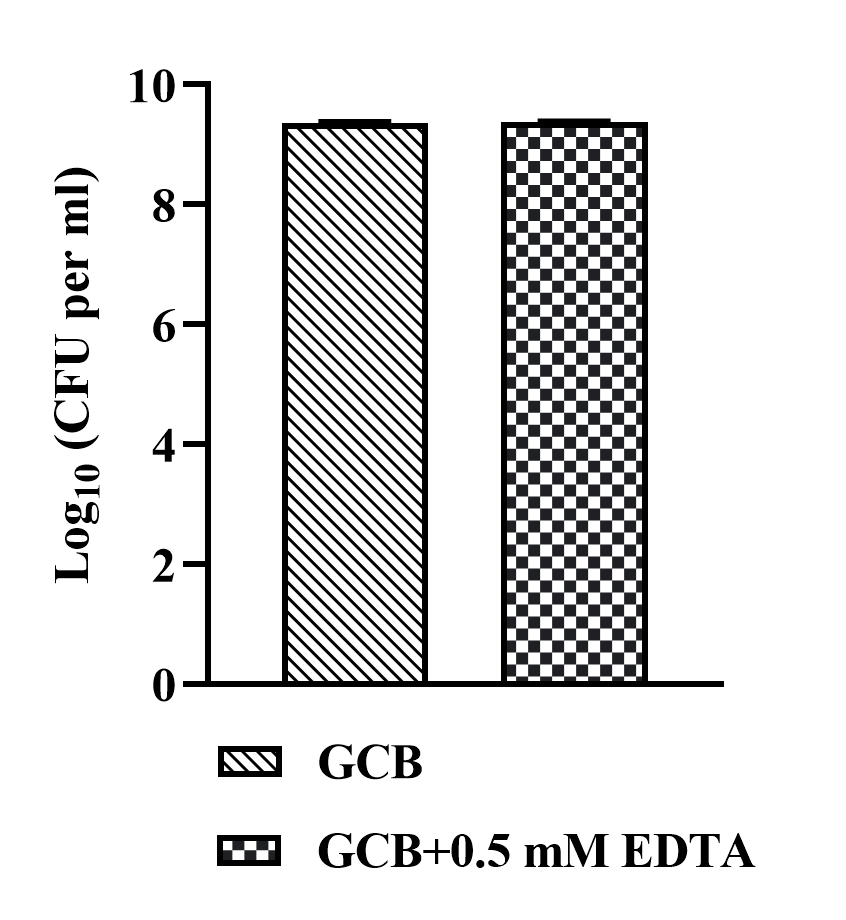


**Supplementary Figure 2 The viable bacteria treated with or without 0.5 mM EDTA.** The bacteria were incubated into GCB with or without 0.5 mM EDTA for 30 minute and diluted bacteria serially to spread on the GCB plates for counting bacteria. Error bars denote standard deviation.

**
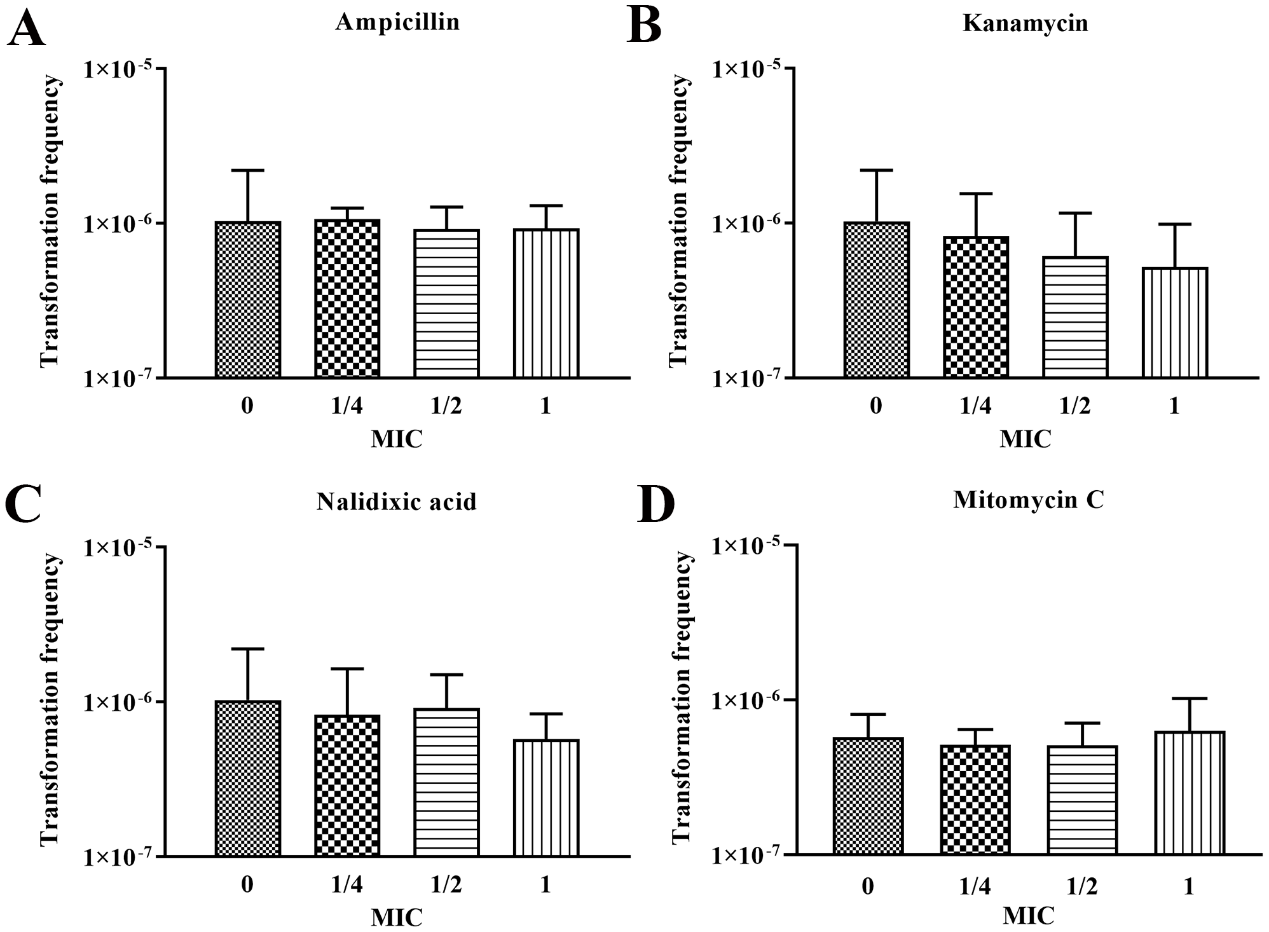
**

**Supplementary Figure 3 The effect of antibiotics on natural transformation of *R. columbina*.** Wild-type cells were treated with different concentrations of ampicillin (A), kanamycin (B), nalidixic acid (C) or mitomycin C (D) for 1 h and then transformed with 1 μg tDNA harboring an antibiotic resistance cassette to calculate the transformation frequency All the results are representative of three independent experiments. Error bars denote standard deviation.
